# Supplementary material for: Stable isotopes of Hawaiian spiders reflect substrate properties along a chronosequence
Source: PeerJ. 2018 Mar 21;6:e4527. doi: 10.7717/peerj.4527 (PMC5866714; doi:10.7717/peerj.4527)
Supplement: Table S1 — Sample sizes of spider species collected at each study site, for (1) Full dataset (used in main paper), (2) Subsampled dataset containing n = 10 for Tetragnatha species whose original sample size was >10 at a given site, (3) Species-controlled dataset excluding the four Tetragnatha species that were not found at all sites (and with T.“golden dome” randomly subsampled to same sample size as T. anuenue). Subsamples used in “Subsampled” and “Sp.-controlled” datasets were taken randomly. Substrate ages are: Upper Waiakea: 200–750 y; 'Ola'a: 2,100 y; Laupāhoehoe: 20,000 y. [file peerj-06-4527-s001.docx]

| Species | Functional group | Site | Sample size: full dataset | Sample size: subsampled | Sample size: sp.-controlled |
| --- | --- | --- | --- | --- | --- |
| *Tetragnatha anuenue* | Spiny Leg | Upper Waiakea | 9 | 9 | 9 |
|  |  | ‘Ola’a | 14 | 10 | 14 |
|  |  | Laupāhoehoe | 12 | 10 | 12 |
| *Tetragnatha brevignatha* | Spiny Leg | Upper Waiakea | 10 | 10 | 0 |
|  |  | ‘Ola’a | 0 | 0 | 0 |
|  |  | Laupāhoehoe | 14 | 10 | 0 |
| *Tetragnatha quasimodo* | Spiny Leg | Upper Waiakea | 12 | 10 | 0 |
|  |  | ‘Ola’a | 1 | 0 | 0 |
|  |  | Laupāhoehoe | 14 | 10 | 0 |
| *Tetragnatha hawaiensis* | web-builder | Upper Waiakea | 20 | 10 | 0 |
|  |  | ‘Ola’a | 7 | 7 | 0 |
|  |  | Laupāhoehoe | 5 | 5 | 0 |
| *Tetragnatha perkinsi* | web-builder | Upper Waiakea | 12 | 10 | 0 |
|  |  | ‘Ola’a | 5 | 5 | 0 |
|  |  | Laupāhoehoe | 0 | 0 | 0 |
| *Tetragnatha* sp. “golden dome” | web-builder | Upper Waiakea | 30 | 10 | 10 |
|  |  | ‘Ola’a | 18 | 10 | 14 |
|  |  | Laupāhoehoe | 28 | 10 | 12 |
| *Ariamnes* spp. (*A. hiwa* + *A. waikula*) | *Ariamnes* (spider eater) | Upper Waiakea | 12 | 12 | 12 |
|  |  | ‘Ola’a | 9 | 9 | 9 |
|  |  | Laupāhoehoe | 9 | 9 | 9 |
