# Supplementary material for: Stable isotopes of Hawaiian spiders reflect substrate properties along a chronosequence
Source: PeerJ. 2018 Mar 21;6:e4527. doi: 10.7717/peerj.4527 (PMC5866714; doi:10.7717/peerj.4527)
Supplement: Table S2 — Tukey’s HSD test comparing effects of site (substrate) on carbon and nitrogen stable isotopes of different functional groups. Signficant differences are indicated in bold. Functional groups are included only when results of preliminary ANOVA show a significant effect of site. Substrate ages are: Upper Waiakea: 200–750 y; 'Ola'a: 2,100 y; Laupāhoehoe: 20,000 y. [file peerj-06-4527-s002.docx]

| **Isotope** | **Functional group** | **Comparison** | **Tukey’s adjusted p-value** |
| --- | --- | --- | --- |
| δ^15^N | plants | Upper Waiakea:’Ola’a | **< 0.001** |
|  |  | ‘Ola’a:Laupāhoehoe | **0.012** |
|  |  | Upper Waiakea:Laupāhoehoe | **< 0.001** |
|  | Spiny Leg | Upper Waiakea:’Ola’a | **< 0.001** |
|  |  | ‘Ola’a:Laupāhoehoe | **< 0.001** |
|  |  | Upper Waiakea:Laupāhoehoe | **< 0.001** |
|  | web-builders | Upper Waiakea:’Ola’a | **< 0.001** |
|  |  | ‘Ola’a:Laupāhoehoe | **< 0.001** |
|  |  | Upper Waiakea:Laupāhoehoe | **< 0.001** |
|  | Ariamnes | Upper Waiakea:’Ola’a | **< 0.001** |
|  |  | ‘Ola’a:Laupāhoehoe | 0.203 |
|  |  | Upper Waiakea:Laupāhoehoe | **< 0.001** |
| δ^13^C | Spiny Leg | Upper Waiakea:’Ola’a | 0.120 |
|  |  | ‘Ola’a:Laupāhoehoe | **< 0.005** |
|  |  | Upper Waiakea:Laupāhoehoe | 0.251 |
|  | web-builders | Upper Waiakea:’Ola’a | **< 0.001** |
|  |  | ‘Ola’a:Laupāhoehoe | **< 0.001** |
|  |  | Upper Waiakea:Laupāhoehoe | **< 0.005** |
|  | Ariamnes | Upper Waiakea:’Ola’a | **< 0.001** |
|  |  | ‘Ola’a:Laupāhoehoe | **< 0.001** |
|  |  | Upper Waiakea:Laupāhoehoe | **0.008** |
