# Supplementary material for: Stable isotopes of Hawaiian spiders reflect substrate properties along a chronosequence
Source: PeerJ. 2018 Mar 21;6:e4527. doi: 10.7717/peerj.4527 (PMC5866714; doi:10.7717/peerj.4527)
Supplement: Table S3 — Tukey’s HSD test comparing stable isotopes of pairs of functional groups within each site. Signficant differences are indicated in bold. Plant leaves (“plants”) were consistently found to be significantly lower in both δ13C and δ15N than any functional group of spiders, but were closest to Spiny Leg spiders; therefore, comparisons “plants:web-builders” and “plants:Ariamnes” are not shown. [file peerj-06-4527-s003.docx]

| **Isotope** | **Site** | **Comparison** | **Tukey’s adjusted p-value** |
| --- | --- | --- | --- |
| δ^15^N | Upper Waiakea  (200-750 y) | plants:Spiny Leg | **< 0.001** |
|  |  | Spiny Leg:web-builders | **< 0.001** |
|  |  | web-builders:Ariamnes | 0.071 |
|  |  | plants:leaf litter | **< 0.001** |
|  |  | Spiny Leg:leaf litter | **0.008** |
|  |  | web-builders:leaf litter | 0.998 |
|  |  | Ariamnes:leaf litter | 0.512 |
|  | ‘Ola’a  (2,100 y) | plants:Spiny Leg | **< 0.001** |
|  |  | Spiny Leg:web-builders | **< 0.001** |
|  |  | web-builders:Ariamnes | **< 0.001** |
|  | Laupāhoehoe  (20,000 y) | plants:Spiny Leg | **< 0.001** |
|  |  | Spiny Leg:web-builders | **< 0.001** |
|  |  | Spiny Leg:Ariamnes | **< 0.001** |
|  |  | web-builders:Ariamnes | **< 0.001** |
| δ^13^C | Upper Waiakea  (200 – 750 y) | plants:leaf litter | 0.140 |
|  |  | plants:Spiny Leg | **< 0.001** |
|  |  | leaf litter:Spiny Leg | **0.002** |
|  |  | Spiny Leg:web-builders | **< 0.001** |
|  |  | Spiny Leg:Ariamnes | 0.076 |
|  |  | web-builders:Ariamnes | 0.999 |
|  | ‘Ola’a  (2,100 y) | plants:Spiny Leg | **< 0.001** |
|  |  | Spiny Leg:web-builders | **< 0.001** |
|  |  | Spiny Leg:Ariamnes | **< 0.001** |
|  |  | web-builders:Ariamnes | 0.940 |
|  | Laupāhoehoe  (20,000 y) | plants:Spiny Leg | **< 0.001** |
|  |  | Spiny Leg:web-builders | 0.053 |
|  |  | Spiny Leg:Ariamnes | 0.442 |
|  |  | web-builders:Ariamnes | 0.999 |
