# Supplementary material for: Stable isotopes of Hawaiian spiders reflect substrate properties along a chronosequence
Source: PeerJ. 2018 Mar 21;6:e4527. doi: 10.7717/peerj.4527 (PMC5866714; doi:10.7717/peerj.4527)
Supplement: Table S4 — Side-by-side comparisons of results of 2-way ANOVA with site and functional group as factors, showing statistics for: (1) Full dataset (used in main paper), (2) Subsampled dataset, and (3) Species-controlled dataset (see Table S1 for sample sizes of the three datasets). Significance does not change under different subsampling regimes. [file peerj-06-4527-s004.docx]

| Isotope | Effect | all data | | | subsampled | | | sp.-controlled | | |
| --- | --- | --- | --- | --- | --- | --- | --- | --- | --- | --- |
|  |  | F | df | p-value | F | df | p-value | F | df | p-value |
| δ^15^N | site | 692.1 | 2 | **< 0.001** | 589.6 | 2 | **< 0.001** | 435.0 | 2 | **< 0.001** |
|  | functional group | 113.6 | 4 | **< 0.001** | 101.6 | 4 | **< 0.001** | 112.2 | 4 | **< 0.001** |
|  | site:functional group | 8.615 | 6 | **< 0.001** | 5.527 | 6 | **< 0.001** | 8.199 | 6 | **< 0.001** |
| δ^13^C | site | 55.51 | 2 | **< 0.001** | 57.21 | 2 | **< 0.001** | 49.44 | 2 | **< 0.001** |
|  | functional group | 95.15 | 4 | **< 0.001** | 91.56 | 4 | **< 0.001** | 96.79 | 4 | **< 0.001** |
|  | site:functional group | 1.841 | 6 | 0.092 | 1.396 | 6 | 0.219 | 0.6937 | 6 | 0.655 |
