# Supplementary material for: Stable isotopes of Hawaiian spiders reflect substrate properties along a chronosequence
Source: PeerJ. 2018 Mar 21;6:e4527. doi: 10.7717/peerj.4527 (PMC5866714; doi:10.7717/peerj.4527)
Supplement: Table S5 — Side-by-side comparisons of results of ANOVA testing for effects of site within functional group, showing statistics for: (1) Full dataset (used in main paper), (2) Subsampled dataset, and (3) Species-controlled dataset (see Table S1 for sample sizes of the three datasets). Significance does not change under different subsampling regimes. [file peerj-06-4527-s005.docx]

| Isotope | Comparison | all data | | | subsampled | | | sp.-controlled | | |
| --- | --- | --- | --- | --- | --- | --- | --- | --- | --- | --- |
|  |  | F | df | p-value | F | df | p-value | F | df | p-value |
| δ^15^N | plants | 78.74 | 2 | **< 0.001** | 78.74 | 2 | **< 0.001** | 78.74 | 2 | **< 0.001** |
|  | Spiny Leg | 446.9 | 2 | **< 0.001** | 338.2 | 2 | **< 0.001** | 161.8 | 2 | **< 0.001** |
|  | web-builders | 216.6 | 2 | **< 0.001** | 123.5 | 2 | **< 0.001** | 83.93 | 2 | **< 0.001** |
|  | *Ariamnes* | 80.87 | 2 | **< 0.001** | 76.13 | 2 | **< 0.001** | 76.13 | 2 | **< 0.001** |
| δ^13^C | plants | 0.7997 | 2 | 0.482 | 0.7997 | 2 | 0.482 | 0.7997 | 2 | 0.482 |
|  | Spiny Leg | 5.681 | 2 | **0.005** | 5.392 | 2 | **0.007** | 14.24 | 2 | **< 0.001** |
|  | web-builders | 31.91 | 2 | **< 0.001** | 29.46 | 2 | **< 0.001** | 18.33 | 2 | **< 0.001** |
|  | *Ariamnes* | 36.62 | 2 | **< 0.001** | 40.85 | 2 | **< 0.001** | 40.85 | 2 | **< 0.001** |
