# Supplementary material for: Stable isotopes of Hawaiian spiders reflect substrate properties along a chronosequence
Source: PeerJ. 2018 Mar 21;6:e4527. doi: 10.7717/peerj.4527 (PMC5866714; doi:10.7717/peerj.4527)
Supplement: Table S6 — Side-by-side comparisons of results of ANOVA testing for effects of functional group within site, showing statistics for: (1) Full dataset (used in main paper), (2) Subsampled dataset, and (3) Species-controlled dataset (see Table S1 for sample sizes of the three datasets). Significance does not change under different subsampling regimes. [file peerj-06-4527-s006.docx]

| Isotope | Site | all data | | | subsampled | | | sp.-controlled | | |
| --- | --- | --- | --- | --- | --- | --- | --- | --- | --- | --- |
|  |  | F | df | p-value | F | df | p-value | F | df | p-value |
| δ^15^N | Upper Waiakea (200-750 y) | 68.38 | 4 | **< 0.001** | 61.25 | 4 | **< 0.001** | 88.81 | 4 | **< 0.001** |
|  | ‘Ola’a (2,100 y) | 34.23 | 3 | **< 0.001** | 26.15 | 3 | **< 0.001** | 32.23 | 3 | **< 0.001** |
|  | Laupāhoehoe (20,000 y) | 28.90 | 3 | **< 0.001** | 32.87 | 3 | **< 0.001** | 27.43 | 3 | **< 0.001** |
| δ^13^C | Upper Waiakea (200-750 y) | 36.42 | 4 | **< 0.001** | 40.12 | 4 | **< 0.001** | 35.07 | 4 | **< 0.001** |
|  | ‘Ola’a (2,100 y) | 41.29 | 3 | **< 0.001** | 33.00 | 3 | **< 0.001** | 35.20 | 3 | **< 0.001** |
|  | Laupāhoehoe (20,000 y) | 41.48 | 3 | **< 0.001** | 37.11 | 3 | **< 0.001** | 54.38 | 3 | **< 0.001** |
