# Supplementary material for: Stable isotopes of Hawaiian spiders reflect substrate properties along a chronosequence
Source: PeerJ. 2018 Mar 21;6:e4527. doi: 10.7717/peerj.4527 (PMC5866714; doi:10.7717/peerj.4527)
Supplement: Table S7 — Side-by-side comparisons of Tukey’s HSD results for effects of site within functional group, showing statistics for: (1) Full dataset (used in main paper), (2) Subsampled dataset, and (3) Species-controlled dataset (see for Table S1 sample sizes of the three datasets). With rare exception, significance does not change under different subsampling regimes. [file peerj-06-4527-s007.docx]

| isotope | functional group | comparison | Tukey’s adjusted p-values | | |
| --- | --- | --- | --- | --- | --- |
|  |  |  | all data | subsampled | sp.-controlled |
| δ^15^N | plants | Upper Waiakea:’Ola’a | **< 0.001** | **< 0.001** | **< 0.001** |
|  |  | ‘Ola’a:Laupāhoehoe | **0.012** | **0.012** | **0.012** |
|  |  | Upper Waiakea:Laupāhoehoe | **< 0.001** | **< 0.001** | **< 0.001** |
|  | Spiny Leg | Upper Waiakea:’Ola’a | **< 0.001** | **< 0.001** | **< 0.001** |
|  |  | ‘Ola’a:Laupāhoehoe | **< 0.001** | **< 0.001** | **< 0.001** |
|  |  | Upper Waiakea:Laupāhoehoe | **< 0.001** | **< 0.001** | **< 0.001** |
|  | web-builders | Upper Waiakea:’Ola’a | **< 0.001** | **< 0.001** | **< 0.001** |
|  |  | ‘Ola’a:Laupāhoehoe | **< 0.001** | **< 0.001** | **< 0.001** |
|  |  | Upper Waiakea:Laupāhoehoe | **< 0.001** | **< 0.001** | **< 0.001** |
|  | Ariamnes | Upper Waiakea:’Ola’a | **< 0.001** | **< 0.001** | **< 0.001** |
|  |  | ‘Ola’a:Laupāhoehoe | 0.203 | 0.185 | 0.185 |
|  |  | Upper Waiakea:Laupāhoehoe | **< 0.001** | **< 0.001** | **< 0.001** |
| δ^13^C | Spiny Leg | Upper Waiakea:’Ola’a | 0.120 | 0.086 | **0.006** |
|  |  | ‘Ola’a:Laupāhoehoe | **0.004** | **0.005** | **< 0.001** |
|  |  | Upper Waiakea:Laupāhoehoe | 0.251 | 0.286 | 0.359 |
|  | web-builders | Upper Waiakea:’Ola’a | **< 0.001** | **< 0.001** | **0.017** |
|  |  | ‘Ola’a:Laupāhoehoe | **< 0.001** | **< 0.001** | **< 0.001** |
|  |  | Upper Waiakea:Laupāhoehoe | **< 0.001** | **0.012** | **0.026** |
|  | Ariamnes | Upper Waiakea:’Ola’a | **< 0.001** | **< 0.001** | **< 0.001** |
|  |  | ‘Ola’a:Laupāhoehoe | **< 0.001** | **< 0.001** | **< 0.001** |
|  |  | Upper Waiakea:Laupāhoehoe | **0.008** | **0.019** | **0.019** |
