# Supplementary material for: Stable isotopes of Hawaiian spiders reflect substrate properties along a chronosequence
Source: PeerJ. 2018 Mar 21;6:e4527. doi: 10.7717/peerj.4527 (PMC5866714; doi:10.7717/peerj.4527)
Supplement: Table S8 — Side-by-side comparisons of Tukey’s HSD results for effects of functional group within site, showing statistics for: (1) Full dataset (used in main paper), (2) Subsampled dataset, and (3) Species-controlled dataset (see Table S1 for sample sizes of the three datasets). With rare exception, significance does not change under different subsampling regimes. [file peerj-06-4527-s008.docx]

| isotope | site | comparison | Tukey’s adjusted p-values | | |
| --- | --- | --- | --- | --- | --- |
|  |  |  | all data | subsampled | sp.-controlled |
| δ^15^N | Upper Waiakea  (200-750 y) | plants:Spiny Leg | **< 0.001** | **< 0.001** | **< 0.001** |
|  |  | Spiny Leg:web-builders | **< 0.001** | **< 0.001** | **< 0.001** |
|  |  | web-builders:Ariamnes | 0.071 | 0.161 | 0.619 |
|  |  | plants:leaf litter | **< 0.001** | **< 0.001** | **< 0.001** |
|  |  | Spiny Leg:leaf litter | **0.008** | **0.006** | **< 0.001** |
|  |  | web-builders:leaf litter | 0.998 | 0.9998 | 0.901 |
|  |  | Ariamnes:leaf litter | 0.512 | 0.598 | 0.364 |
|  | ‘Ola’a  (2,100 y) | plants:Spiny Leg | **< 0.001** | **0.001** | **< 0.001** |
|  |  | Spiny Leg:web-builders | **< 0.001** | **0.014** | **0.017** |
|  |  | web-builders:Ariamnes | **< 0.001** | **0.003** | **< 0.001** |
|  | Laupāhoehoe  (20,000 y) | plants:Spiny Leg | **< 0.001** | **< 0.001** | **< 0.001** |
|  |  | Spiny Leg:web-builders | **< 0.001** | **< 0.001** | **0.012** |
|  |  | Spiny Leg:Ariamnes | **< 0.001** | **< 0.001** | **< 0.001** |
|  |  | web-builders:Ariamnes | **< 0.001** | **0.006** | 0.054 |
| δ^13^C | Upper Waiakea  (200-750 y) | plants:leaf litter | 0.140 | 0.068 | 0.077 |
|  |  | plants:Spiny Leg | **< 0.001** | **< 0.001** | **< 0.001** |
|  |  | leaf litter:Spiny Leg | **0.002** | **< 0.001** | 0.072 |
|  |  | Spiny Leg:web-builders | **< 0.001** | **0.002** | **0.003** |
|  |  | Spiny Leg:Ariamnes | 0.076 | 0.085 | **0.007** |
|  |  | web-builders:Ariamnes | 0.999 | 0.999 | 0.999 |
|  | ‘Ola’a  (2,100 y) | plants:Spiny Leg | **< 0.001** | **< 0.001** | **< 0.001** |
|  |  | Spiny Leg:web-builders | **< 0.001** | **0.003** | **0.013** |
|  |  | Spiny Leg:Ariamnes | **< 0.001** | **0.010** | **< 0.001** |
|  |  | web-builders:Ariamnes | 0.940 | 0.995 | 0.487 |
|  | Laupāhoehoe  (20,000 y) | plants:Spiny Leg | **< 0.001** | **< 0.001** | **< 0.001** |
|  |  | Spiny Leg:web-builders | 0.053 | 0.274 | **< 0.001** |
|  |  | Spiny Leg:Ariamnes | 0.442 | 0.370 | **< 0.001** |
|  |  | web-builders:Ariamnes | 0.999 | 0.999 | 0.999 |
