# Supplementary figures and images for: Stable isotopes of Hawaiian spiders reflect substrate properties along a chronosequence
Source: PeerJ. 2018 Mar 21;6:e4527. doi: 10.7717/peerj.4527 (PMC5866714; doi:10.7717/peerj.4527)

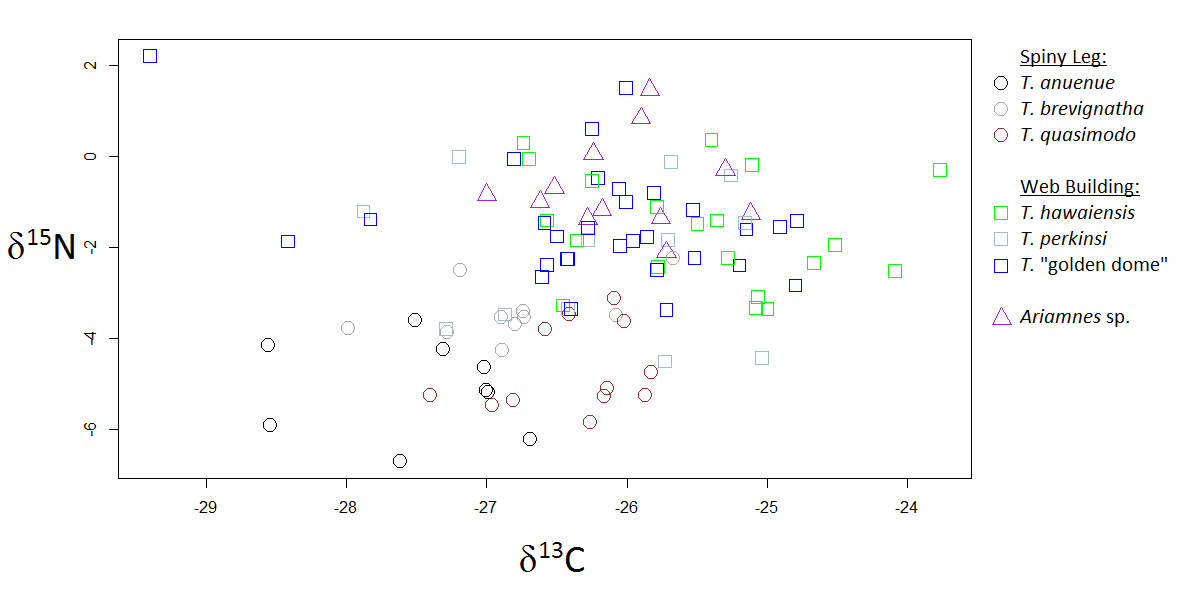

Supplement: Figure S1 — Biplot showing C and N stable isotope signatures of all spider specimens collected from Upper Waiakea (200–750 y). [file peerj-06-4527-s013.png]

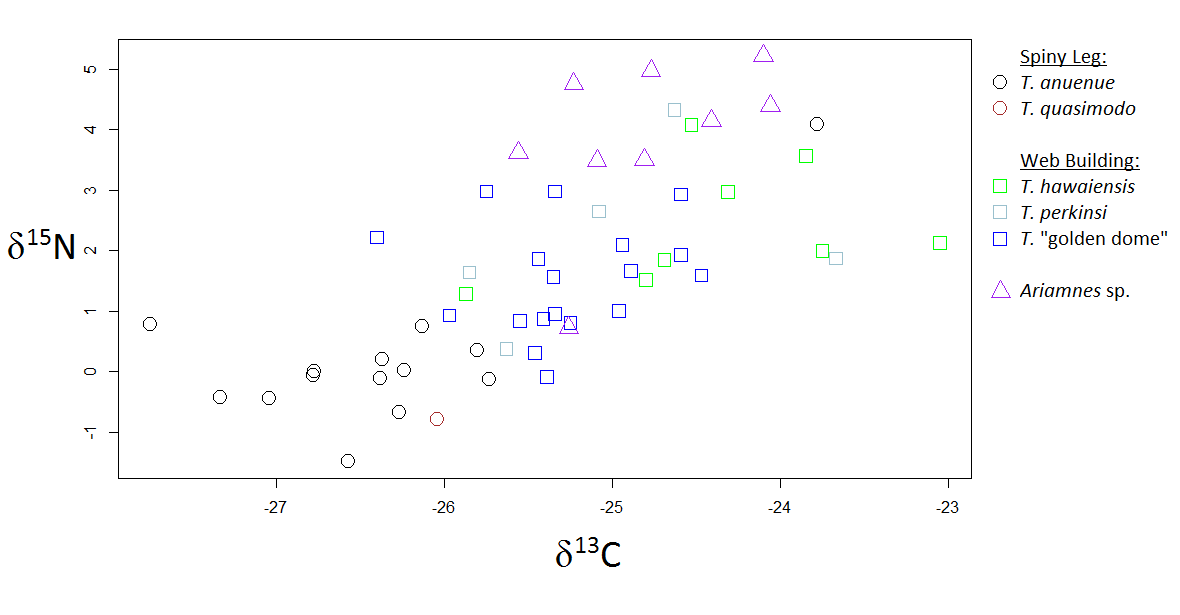

Supplement: Figure S2 — Biplot showing C and N stable isotope signatures of all spider specimens collected from 'Ola'a (2,100 y). [file peerj-06-4527-s014.png]

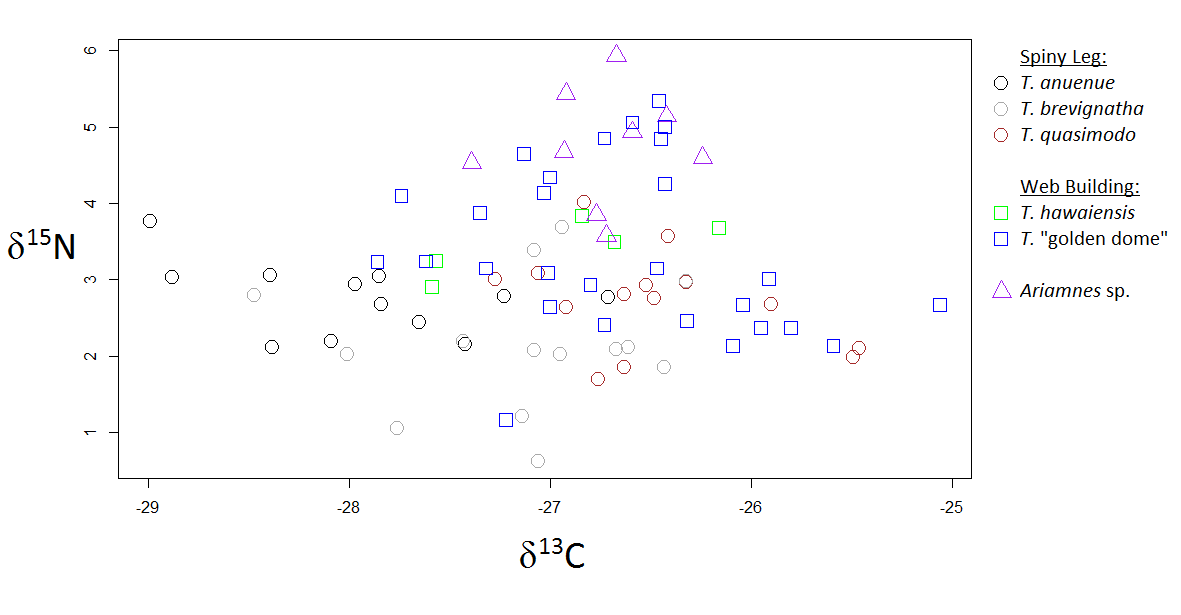

Supplement: Figure S3 — Biplot showing C and N stable isotope signatures of all spider specimens collected from Laupahoehoe (20,000 y). [file peerj-06-4527-s015.png]
